# Loneliness and depressive symptoms among men who have sex with men in China: A cross-sectional study

**DOI:** 10.3389/fpsyt.2023.1179703

**Published:** 2023-04-06

**Authors:** Yuhan Liu, Yuting Yang, Chaowei Fu, Haijiang Lin, Tingting Wang, Shanling Wang, Jiawen Kuang, Xiaoxiao Chen, Jingyi Wang

**Affiliations:** ^1^NHC Key Laboratory of Health Technology Assessment, School of Public Health, Fudan University, Shanghai, China; ^2^Taizhou City Center for Disease Prevention and Control, Taizhou, China

**Keywords:** MSM, loneliness, depression, social support, self-esteem

## Abstract

**Background:**

While psychosocial problems and their related factors in men who have sex with men (MSM) have been well documented in developed countries, there are still not many studies addressing this issue in China and the results are inconsistent. This study aimed to assess the prevalence of loneliness and depressive symptoms among MSM, examine their associated factors, and investigate potential factors moderating the link between depressive symptoms and loneliness.

**Methods:**

A cross-sectional study was conducted in Taizhou of Zhejiang Province in China between April and November 2021. Loneliness was assessed using the 3-item UCLA Loneliness Scale (UCLA-3), and depressive symptoms were measured using the Patient Health Questionaire-9 (PHQ-9). Data from 655 MSM were eligible for analysis. Logistic regression models were used to examine the associations between independent variables and the outcomes of loneliness and depression. The interaction terms were added in the models to assess the moderating effects.

**Results:**

Of the MSM sample, 13.28 and 7.48% perceived loneliness and reported moderate-to-severe depressive symptoms, respectively. We found that participants who experienced loneliness were more likely to have younger age (OR 0.44, 95% CI 0.21, 0.93, 15–32 years as reference group), low social support (OR 3.60, 95% CI 2.14, 6.04), low self-esteem (OR 3.03, 95% CI 1.45, 6.32) and moderate-to-severe depressive symptoms (OR 5.45, 95% CI 2.66, 11.15). The participants with moderate-to-severe depressive symptoms were more likely to have low self-esteem (OR 6.78, 95% CI 3.08, 14.95) and feelings of loneliness (OR 5.51, 95% CI 2.66, 11.40). Stratified analyzes showed that the magnitude of the associations between depressive symptoms and loneliness varied in MSM with different age, marital status, and self-esteem.

**Conclusion:**

Our study suggests that we need to pay attention to feelings of loneliness and depressive symptoms and their closely associated factors such as social support and self-esteem among MSM in China. The MSM who were young, not married, and had low self-esteem were especially vulnerable to the impact of depressive symptoms on loneliness.

## 1. Introduction

As a minority group discriminated by mainstream society, men who have sex with men (MSM) experience complex and multiple psychosocial challenges. The existing evidence in the literature has shown higher prevalence of psychosocial problems such as loneliness (1, 2) and depression (3, 4) among MSM than the general population. MSM are stigmatized due to their sexual orientation, which makes them become isolated and lonely and highly vulnerable to mental health problems (5). A meta-analysis reported high pooled lifetime prevalence of suicidal ideation (34.97%) among MSM (6). The risk of suicidality among MSM has been found to be magnified by a synergistic effect of psychosocial problems (7).

Loneliness is an unpleasant, subjective experience that results from the discrepancy between desired and actual social relationships (2). In the general population, loneliness is a serious health concern that increases the risk of distress (depression and anxiety), suicidal thoughts, unhealthy behaviors, and use of medical services (8). MSM population may feel more helpless and lonelier due to disapproval, discrimination and even exclusion by families and friends. In a study among young black MSM, approximately 19% of the participants reported distressing and bothersome loneliness (9). Marziali et al. (10) found that 61% of the MSM in Vancouver, Canada experienced loneliness, and 87% of MSM in poor health were lonely. Higher levels of loneliness in MSM are associated with negative outcomes such as depression, anxiety, and sexual impulsivity (11). Loneliness is also associated with risky sexual behaviors, substance use, and HIV infection (12, 13).

Depression is another psychosocial and mental health problem in MSM. There is increasing evidence that the prevalence of depression among MSM is higher than that of non-MSM in many parts of the world (14). In a meta-analysis, the pooled prevalence of depression was 35% among MSM and 47% among those living with HIV (15). Discrimination, stigma, and minority stress toward MSM may play a major role in the development of depression (16). Similar to loneliness, depression is associated with poor sexual functioning, condomless sex, substance use, and reduced use of HIV prevention services and care (17–20). Many studies in the general population have shown that there is an association between loneliness and depression (21). Some studies found that loneliness increased the risk of developing depressive symptoms (8, 22), while others have proposed a two-way, reciprocal relationship in which those who are depressed are more likely to feel lonely and those who are lonely may experience depression as a result of lack of social contact (23, 24). Cacioppo et al. (24) also reported that loneliness and depressive symptoms can act in a synergistic way to impair well-being in middle-aged and older adults (25). However, the relationship between loneliness and depression and their associated factors in MSM have not been fully established.

Depression, as well as loneliness, has also been related to social support and self-esteem. Social support was associated with increased access to medical service use (26). Individuals with insufficient social support would have a greater risk of experiencing the negative impact of stress. A systematic review of the association between social relationships and depression highlighted the significant protective effects of perceived emotional support and perceived instrumental support (27). In another review, less emotional or social support was also related to loneliness (28). This is particularly relevant to lonely MSM with small social networks, who may get limited access to social support and community resources (29). In addition to external deterrents, there are ‘self-issues’ such as self-esteem, and internalized stigma which negatively affect social participation, disclosure of sexual orientation and access to health care (30, 31). Self-esteem is an individual’s own evaluation of their personal worth which is often negatively affected by perceived and internalized stigma (32). There is existing evidence of the relationships between self-esteem and depression, anxiety, loneliness, and life satisfaction (33–35). Therefore, social support and self-esteem are important constructs which researchers need to take into account when studying psychosocial problems in MSM.

While psychosocial problems and their related factors in MSM have been well documented in developed countries, there are still not many studies addressing this issue in China. The situation might be even more serious in China as the MSM population may not be accepted by most Chinese families. One study in China reported the high prevalence and associated factors of depression and loneliness in MSM (2). However, it only used a single item to measure loneliness and did not examine moderators of the association between depression and loneliness. In the past few years, China has taken behavioral interventions and condom promotion to reduce the sexual risk of MSM but they have not brought sustained prevention success (36). Therefore, it is very important to explore factors associated with loneliness and depressive symptoms in MSM as sustainable behavioral change will be difficult to achieve if psychosocial and mental health factors are not considered.

In this study, we sought to advance the understanding of psychosocial problems in MSM through an investigation of patterns of loneliness and depression among a diverse sample of MSM in Taizhou, China. Taizhou, located on the coast of eastern China and central Zhejiang Province, has about 6 million residents (37). In terms of population scale and economic development, Taizhou is a medium-sized city in China and the gross domestic product is at an intermediate level among all the cities in Zhejiang Province. Taizhou consists of three districts (Jiaojiang, Huangyan, and Luqiao), three county-level cities (Linhai, Wenling, and Yuhuan) and three counties (Tiantai, Xianju, and Sanmen). A previous study suggested that the prevalence of risky sexual behaviors among MSM in this city was higher than in many other areas in China (38). Thus, they represent an ideal group for researchers to begin to understand the extent of and the factors associated with loneliness and depression in MSM population. Our study aimed to assess the prevalence of loneliness and depressive symptoms among MSM. We also aimed to examine their associations with demographic factors, psychosocial conditions, and risky sexual behaviors, and to investigate potential factors moderating the link between depressive symptoms and loneliness.

## 2. Materials and methods

### 2.1. Participants

A cross-sectional study was performed from April to November 2021 among MSM in three districts: Huangyan, Jiaojiang, and Luqiao in Taizhou of Zhejiang Province. To be eligible for the study, participants had to meet the following criteria: (1) be biologically male; (2) be at least 15 years of age; (3) have had oral or anal sex with another male in the past; (4) don’t have communication problems or severe cognitive impairment; and (5) be willing to complete the questionnaire.

As MSM is a less accessible group, we applied a combination of convenience sampling and snowball sampling to recruit participants. Firstly, the questionnaires were sent out in venues frequented by MSM such as bars, nightclubs, sauna rooms, parks, and so on. The participants were asked to complete the questionnaire on their own in a private area of the venue in order to protect individual privacy, and the trained researchers were available to provide clarity if participants were unsure how to reply to any questions. At the same time, we posted recruitment information on the most popular provincial gay-oriented web forums, chat rooms, and chat apps. Then the primary participants were encouraged to pass the questionnaire on to other MSM until we got enough samples. A total of 658 MSM provided written informed consent and completed questionnaires. After removing the cases with missing values, we finally analyzed data derived from 655 individuals who participated in the study.

### 2.2. Measures

#### 2.2.1. Outcome variables

##### 2.2.1.1. Loneliness

The 3-item UCLA Loneliness Scale (UCLA-3) (39) was used to assess feelings of loneliness. Each item asked participants to indicate how often they feel this way in general and was answered on a 3-point scale (1 = hardly ever; 2 = some of the time; 3 = often). Scores of the scale range from 3 to 9, with higher scores indicating higher levels of perceived loneliness. Those who had scores of 6 or above were classified as having loneliness in our study. This scale’s reliability and validity have been proven (40, 41) and the Cronbach’s alpha was 0.76 in this research.

##### 2.2.1.2. Depressive symptoms

The Chinese version of Patient Health Questionaire-9 (PHQ-9) was used to measure depressive symptoms within the past 2 weeks. The PHQ-9 has 9 items which were rated on a 4-point Likert scale ranging from 0 (not at all) to 3 (nearly every day). The possible total score ranges from 0 to 27, with higher scores indicating more severe depressive symptoms. Those who had scores of 10 or above were classified as having moderate-to-severe depressive symptoms in our study. PHQ-9 has been demonstrated as a valid and reliable tool to screen depression in the general Chinese population (42), and the Cronbach’s alpha was 0.88 in this study.

#### 2.2.2. Independent variables

Participants were asked about their age, educational level, marital status, sexual orientation, whether or not they had engaged in intercourse with women in the past 6 months, whether or not they were tested for sexually transmitted diseases (STDs) in the last year and the results of the most recent HIV test.

##### 2.2.2.1. Risky sexual behaviors

Four items were chosen to quantify the participants’ risky sexual behaviors (“How often have you used condoms for anal sex in the last 6 months?”, “How many times have you searched for MSM individuals over the Internet and had temporary sex with them in the last 6 months?”, “How often have you used condoms for temporary sex with the above individuals in the last 6 months?”, “How often have you used condoms for commercial sex with men in the last 6 months?”). The second item was answered with average times per month. The rest items were answered on a 3-point scale (1 = every time; 2 = some of the time; 3 = never. If no corresponding behavior occurred, the item scored 0). Higher total scores of the four items indicate greater sexual risk.

##### 2.2.2.2. Social support

The Chinese version of the Multidimensional Scale of Perceived Social Support (MSPSS) was used to measure participants’ social support (43). The MSPSS consists of 12 items rated on a 7-point Likert scale ranging from 1 (strongly disagree) to 7 (strongly agree), with higher scores indicating higher social support. The total scores were divided into three equal groups and the lowest group was considered as low social support. The Cronbach’s alpha was 0.93 in this study.

##### 2.2.2.3. Self-esteem

The Chinese version of the Rosenberg Self-Esteem Scale (RSES) was used to measure participants’ self-esteem (43). The scale is a ten-item measure. The score for each item ranges from 1 (strongly disagree) to 4 (strongly agree). Items 3, 5, 8, 9, and 10 were reversely coded. A higher total score indicates a greater level of self-esteem, and a score below 15 suggests low self-esteem. The Cronbach’s alpha was 0.78 in this study.

### 2.3. Statistical analysis

Descriptive analyzes were conducted to describe the characteristics of the sample. We presented categorical variables as frequencies and percentages, and the continuous variable as median and interquartile. The internal consistency of the scales was assessed using Cronbach’s alpha coefficients.

Bivariate logistic regression analyzes were used to examine the associations between independent variables and outcomes of loneliness and depression. Variables that were associated with loneliness or depression at *p* < 0.20 in bivariate analysis were introduced into multivariable logistic regression models to control for possible confounding factors. Odds ratio (95% CI) and *p*-values were reported.

To assess moderating effects of the factors in the multivariable models for loneliness, interactions between the independent variables (age, education, marital status, and intercourse with women in the past 6 months, risky sexual behaviors, social support, and self-esteem) and depressive symptoms were tested. The interaction terms of age, marital status, self-esteem and depressive symptoms were significant. Therefore, stratified analyzes were conducted by age (14–32 years, 33–83 years, divided according to median age of 32), marital status (never married/previously married, married, or cohabiting), and self-esteem (high, low) in the regression models for the associations between depressive symptoms and loneliness. The interactions between the independent variables and loneliness were also tested for depressive symptoms but no marked interactions were found.

We conducted all analyzes in Stata MP 16 with the level of significance determined at 0.05 *p*-value, and the figure was performed by GraphPad Prism 9 software.

## 3. Results

The socio-demographic characteristics of the participants (*n* = 655) are illustrated in Table 1. Of the participants with a median age of 32 years (range 15–83), 40.31% had a middle school degree or less, 36.34% were married or cohabiting, and 57.40% were self-identified as homosexual. Around 24.43% reported having sexual intercourse with women in the past 6 months, and 20.00% scored above the upper quartile of the total score for risky sexual behaviors (> 75% of data points). The percentage of participants who had sexually transmitted diseases in the last year and were HIV-positive was 2.75 and 3.36%, respectively. Of the participants, 13.28% reported feelings of loneliness, and 7.48% reported moderate-to-severe depressive symptoms. Meanwhile, 34.50% had low social support, and 7.33% low self-esteem.

**TABLE 1:** Characteristics of the study sample and factors related to loneliness and depressive symptoms.

Characteristics	Overall (*N* = 655), (*n*, %)	Loneliness		Depressive symptoms	
		Odds ratio (95% CI)	*p*-value	Odds ratio (95% CI)	*p*-value
**Age (median = 32, IQR = 18)**					
15–32 years	347 (52.98%)	Ref		Ref	
33–83 years	308 (47.02%)	0.38 (0.23, 0.63)	<0.001	0.42 (0.22, 0.80)	0.009
**Education**					
Middle school or less	264 (40.31%)	Ref		Ref	
High school or technical	257 (39.24%)	1.28 (0.76, 2.16)	0.361	2.06 (1.00, 4.24)	0.049
College or above	134 (20.46%)	1.68 (0.93, 3.04)	0.086	2.45 (1.10, 5.46)	0.028
**Marital status**					
Never married	372 (56.79%)	Ref		Ref	
Married or cohabiting	238 (36.34%)	0.43 (0.25, 0.75)	0.003	0.30 (0.14, 0.65)	0.002
Previously married	45 (6.87%)	0.77 (0.31, 1.90)	0.568	0.40 (0.09, 1.70)	0.214
**Sexual orientation**					
Homosexual	376 (57.40%)	Ref		Ref	
Bisexual/Heterosexual/unsure	279 (42.60%)	0.76 (0.47, 1.21)	0.240	0.41 (0.21, 0.81)	0.010
**Intercourse with women in the past 6 months**					
No	495 (75.57%)	Ref		Ref	
Yes	160 (24.43%)	0.36 (0.18, 0.72)	0.004	0.33 (0.13, 0.85)	0.021
**Risky sexual behaviors**					
Below the upper quartile	524 (80.00%)	Ref		Ref	
Above the upper quartile	131 (20.00%)	1.43 (0.84, 2.41)	0.187	1.49 (0.77, 2.90)	0.237
**STDs in the last year**					
No	637 (97.25%)	Ref		Ref	
Yes	18 (2.75%)	1.32 (0.37, 4.64)	0.669	1.57 (0.35, 7.03)	0.556
**HIV positive**					
No	633 (96.64%)	Ref		Ref	
Yes	22 (3.36%)	0.30 (0.04, 2.28)	0.246	2.01 (0.57, 7.06)	0.274
**Social support**					
High	429 (65.50%)	Ref		Ref	
Low	226 (34.50%)	4.49 (2.78, 7.23)	<0.001	3.01 (1.66, 5.46)	<0.001
**Self-esteem**					
High	607 (92.67%)	Ref		Ref	
Low	48 (7.33%)	7.81 (4.19, 14.55)	<0.001	14.24 (7.18, 28.22)	<0.001
**Loneliness**					
No	568 (86.72%)	—		Ref	
Yes	87 (13.28%)	—	—	10.10 (5.43, 18.78)	<0.001
**Depressive symptoms**					
No-mild	606 (92.52%)	Ref		—	
Moderate-to-severe	49 (7.48%)	10.10 (5.43, 18.78)	<0.001	—	—

CI, confidence interval; IQR, interquartile range; Ref, category of reference; STD, sexually transmitted disease; HIV, human immunodeficiency virus.

Table 1 also presents the factors related to loneliness and moderate-to-severe depressive symptoms among the study participants in univariate logistic regression models. Compared to those who did not feel lonely, participants who reported feelings of loneliness were significantly more likely to be 15–32 years old, never married and not had sexual intercourse with women in the past 6 months. They were also more likely to have moderate-to-severe depressive symptoms, low social support and low self-esteem. Meanwhile, compared to those who were non-to-mild depressed, participants who had experienced moderate-to-severe depressive symptoms were significantly more likely to be aged between 15 and 32 years old, have a high school degree or above, never married, self-identified as gay, and not have intercourse with women in the past 6 months. They were also more likely to feel lonely, and have low social support and low self-esteem.

Table 2 presents the factors associated with loneliness and moderate-to-severe depressive symptoms of the study participants in multivariable logistic regression models. Participants who were aged 33–83 years old had smaller odds of experiencing loneliness compared to those aged between 15 and 32 years old (OR 0.44, 95% CI 0.21, 0.93, 15–32 years as reference group). Participants who reported having low social support (OR 3.60, 95% CI 2.14, 6.04), low self-esteem (OR 3.03, 95% CI 1.45, 6.32), and moderate-to-severe depressive symptoms (OR 5.45, 95% CI 2.66, 11.15) were more likely to experience loneliness. Meanwhile, participants who reported having low self-esteem (OR 6.78, 95% CI 3.08, 14.95) and feelings of loneliness (OR 5.51, 95% CI 2.66, 11.40) were more likely to be moderate-to-severe depressed.

**TABLE 2:** Multivariable logistic regression models of the factors associated with loneliness and depressive symptoms.

Characteristics	Loneliness^a^		Depressive symptoms^a^	
	Odds ratio (95% CI)	*p*-value	Odds ratio (95% CI)	*p*-value
**Age**				
15–32 years	Ref		Ref	
33–83 years	0.44 (0.21, 0.93)	0.031	1.56 (0.62, 3.92)	0.340
**Education**				
Middle school or less	Ref		Ref	
High school or technical	0.88 (0.46, 1.67)	0.693	1.51 (0.64, 3.53)	0.344
College or above	1.10 (0.53, 2.29)	0.794	1.90 (0.71, 5.09)	0.203
**Marital status**				
Never married	Ref		Ref	
Married or cohabiting	1.31 (0.58, 2.98)	0.512	0.51 (0.17, 1.57)	0.241
Previously married	1.89 (0.62, 5.80)	0.263	0.52 (0.10, 2.73)	0.439
Sexual orientation	—	—		
Homosexual	—		Ref	
Bisexual/Heterosexual/unsure	—		0.69 (0.30, 1.55)	0.364
**Intercourse with women in the past 6 months**				
No	Ref		Ref	
Yes	0.53 (0.24, 1.18)	0.119	0.82 (0.26, 2.57)	0.737
Risky sexual behaviors			—	—
Below the upper quartile	Ref		—	
Above the upper quartile	1.18 (0.64, 2.18)	0.590	—	
**Social support**				
	Ref		Ref	
Low	3.60 (2.14, 6.04)	<0.001	1.53 (0.76, 3.09)	0.230
**Self-esteem**				
High	Ref		Ref	
Low	3.03 (1.45, 6.32)	0.003	6.78 (3.08, 14.95)	<0.001
Loneliness	—	—		
No	—		Ref	
Yes	—		5.51 (2.66, 11.40)	<0.001
Depressive symptoms			—	—
No-mild	Ref		—	
Moderate-to-severe	5.45 (2.66, 11.15)	<0.001	—	

CI, confidence interval; Ref, category of reference; —, not included in the final multivariable logistic regression model.

^a^Only variables which were associated with loneliness or depressive symptoms at p < 0.20 in the bivariable model were included in the multivariable model.

**FIGURE 1:**
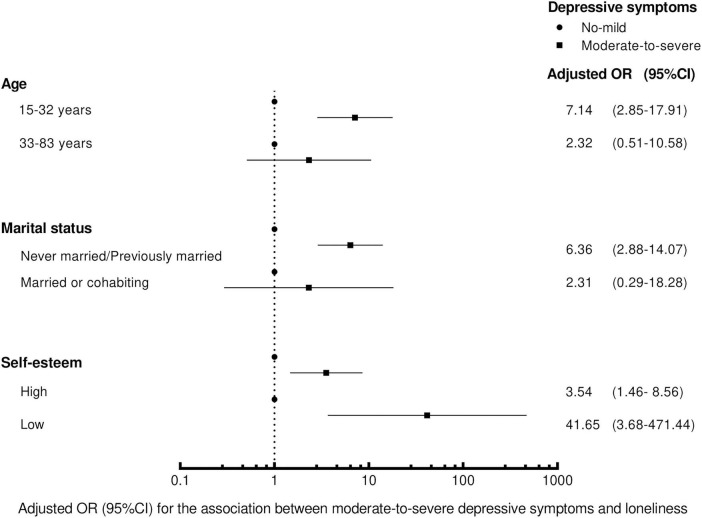
The effect sizes in the association between depressive symptoms and loneliness stratified by age, marital status and self-esteem in logistic regression. The models were adjusted for the variables in Table 2.

In stratified analyzes (Figure 1), we found the magnitude of the associations between depressive symptoms and loneliness varied in MSM with different age (*p* = 0.038), marital status (*p* = 0.086), and self-esteem (*p* = 0.075), when tested as interactions (*p* < 0.1). Compared with MSM having no-to-mild symptoms of depression, those with moderate-to-severe symptoms were more likely to be lonely, and the results were more evident in participants who were aged between 15 and 32 years old (OR = 7.14, 95%CI 2.85–17.91), were never married or previously married (OR = 6.36, 95%CI 2.88–14.07), and had low self-esteem (OR = 41.65, 95%CI 3.68–471.44).

## 4. Discussion

In this study, 13.28% of the participants were classified as feeling lonely and 7.48% were classified as having moderate-to-severe depressive symptoms. The prevalence of loneliness in our sample (13.28%) was similar to young black MSM in Chicago, Illinois (19%) (9), and was slightly higher than the general population in Germany (10.5%) (44). The prevalence of depression in our sample (7.48%) was comparable to the MSM in 12 cities across India (11%) in a study that also used PHQ-9 and the same cut-off score (45), and was a little higher than a nationally representative population of Korea (6.5%) (46). However, our results were lower than some previous studies in Chinese MSM. A study conducted in Jiangsu Province, China found that 35.5% of the participants experienced loneliness which was measured from a single item (2). Among newly diagnosed HIV-positive MSM, the prevalence of moderate-to-severe depression was 41.1% (47). The difference might be attributed to an increased positive public perception of homosexuality in the context of Chinese society. More importantly, the sample population in previous studies was from different regions in China with different characteristics, and different measurement tools were used. Most previous studies reported the prevalence of depression involving mild symptoms and were conducted in HIV-positive MSM. In our study, however, we used a cut-off score for moderate-to-severe depressive symptoms and most of the participants were HIV-negative.

Our study found that loneliness and depressive symptoms were closely associated with each other. The finding coheres with the existing study showing that lonely MSM were more likely to have depressive symptoms (10). They also reported that the effect of loneliness on physical health was partially mediated by depression. A regulatory loop model of loneliness was described by Masi et al. (48). The negative and biased social cognitions adopted by lonely people are likely to get them involved in behavioral confirmation processes and then result in greater loneliness. In people with depression, the negative bias in thinking processes and negative patterns of behavior also interact with one another and form a negative downward loop (49). Therefore, a double feedback loop hypothesis was proposed in which loneliness and depression may reinforce each other and push people further into negative emotions (50). However, whether loneliness causes depression or depression increases the severity of loneliness or both, has not been fully established. More longitudinal studies with long term follow-up in MSM are required to clarify the longitudinal relationship between loneliness and depression.

The results of our study also revealed a high prevalence of other negative psychosocial states including lack of social support (34.50%) and low self-esteem (7.33%), which were significantly associated with loneliness and depression among our sample of MSM participants. Similarly, Wang et al. (51) conducted a survey among MSM in Shanghai and reported that 23.6% of the participants had the low level of social support and 6.6% had low self-esteem. Yan et al. (52) reported close relationships between social support, self-esteem, and depressive symptoms in HIV-positive MSM. Lack of social support and low self-esteem are common among MSM, as they need to hide their sexual orientation or endure discrimination from neighbors, friends, and even family members (53), which may make them feel useless and dissatisfied with themselves. People with low self-esteem may become increasingly withdrawn and self-isolated, which in turn creates a downward spiral of worsening mental illness (52). As a psychosocial problem, low self-esteem can also lead to unprotected anal intercourse, one reason for the spread of HIV and STDs (1). Low social support not only influences mental health, but is also a danger to physical health. MSM with low social support are more likely to report delayed HIV testing and higher HIV virus load (54, 55). Research also shows that social support is related to health of marginalized and stigmatized groups (54). In addition, it is notable that the association between social support and depression in our study was largely weakened after controlling for loneliness, implying that loneliness may mediate their relationship. More studies using mediation analyzes with data from several waves are needed to justify the hypothesis.

Regarding sociodemographic characteristics, MSM with younger age were more likely to experience loneliness and depressive symptoms, although only the association with loneliness remained in the multivariable models. It is consistent with previous population-based surveys which described a U-shaped age distribution of loneliness with higher rates in young people and the elderly (50). Early intervention would be important to prevent lonely young MSM from being stuck in loneliness as they become older (56). Participants who had been married or cohabited with women and those who had intercourse with women in the past 6 months were less likely to feel lonely or have depressive symptoms in univariate models. Those who considered their sexual orientation as bisexual, heterosexual, or unsure were less likely to have depressive symptoms. For homosexual people, exposing their sexual orientation may lead to discrimination and stigma which have been proven to be associated with loneliness and depression (57), while marriage and cohabitation can help to hide it (53). After adjusting for the other confounding variables, marital status, intercourse with women and sexual orientation no longer had significant associations with loneliness or depression. It is possible that the impact of psychosocial problems such as lack of social support and low self-esteem is sufficiently severe that it overrides the factors which might, otherwise, be expected to make a difference in loneliness and depression.

In terms of the moderating effect of age, marital status, and self-esteem on the association between depressive symptoms and loneliness, our results indicated that the relationship was particularly stronger for MSM who were young, not married, or had low self-esteem. Although previous studies have reported the separate effects of these factors on loneliness in MSM, our study adds an important component by suggesting that the combination of these factors and depressive symptoms substantially increased the risk for triggering or precipitating loneliness. There is a potential explanation that MSM with these factors and depressive symptoms share similar interpersonal risk factors, such as stigma. Faced with homosexuality-related stigma, MSM who are young or not married have a greater desire for peer relationships, and low self-esteem and depressive symptoms may exacerbate peer relationship problems, which might result in an increase in their feelings of loneliness (58, 59). Previous studies have shown that loneliness co-develops with self-esteem (60) and depression (61) in adolescence, and that the relationship between loneliness and major depression in adulthood is bidirectional (62). In addition, our finding supports the hypothesis proposed by Wang et al. that the overlapping and reinforcing synergistic effect of psychosocial problems might exist in MSM population (1).

The strengths of our study include the use of valid and reliable loneliness and depression scales, the inclusion of important psychosocial variables and the investigation of moderating effects. However, this study has several limitations. First, the participants were recruited from some specific MSM venues and gay-oriented social media in one Chinese province. Thus, our findings may not be generalizable to MSM who hardly go to these venues, do not use social media, or do not live in Zhejiang Province. Future studies may need multiple recruitment methods and data from diverse areas to obtain a representative sample of MSM in China. Second, questionnaires were filled out by the participants themselves who may conceal or forget some facts, so there is potential bias, such as recall bias and social desirability bias. Third, we used self-designed questions of risky sexual behaviors. Future studies may consider other fully validated scales, such as the revised Sociosexual Orientation Inventory which measures promiscuous behavioral tendency (63). Fourth, as a result of the cross-sectional design, no conclusions with regard to causality can be drawn from this study. Finally, the time of study coincided with the COVID-19 pandemic, which may have influenced the results. However, the data of our study were collected between April and November 2021, a relatively stable period in terms of the infection situation and the government’s pandemic measures in China.

There are a number of implications from our findings. The study highlights the importance of paying sufficient attention to the psychosocial problems of the MSM. It may be helpful for professionals at sexual health clinics and HIV-related services to screen for the severity of loneliness and depression and identify people at risk to prevent negative outcomes and improve their quality of life. Evaluation is also beneficial to show that practitioners are genuinely helping MSM who turn to the services and even a simple question asking service users whether they feel lonely or depressed may make an impact. Special preventive measures and support are needed to address psychosocial problems in MSM, especially for those at younger ages, not married and with low self-esteem, in order to reduce the risk of irreversible consequences such as HIV infection and suicide. Traditional interventions, such as condom promotion strategies, have not been as effective as expected in reducing HIV/STD infection in MSM (64), probably because psychosocial problems such as loneliness, depression, lack of social support and low self-esteem are not addressed well in current interventions. Meanwhile, MSM has long been linked with HIV infection and promiscuity in public perception. The stigma which comes from lack of knowledge may further damage the self-esteem of MSM. Therefore, it’s necessary to improve the living environment of MSM by popularizing related knowledge about homosexuality and healthy sexual behaviors. It is also imperative to raise public awareness of the importance of reducing psychosocial problems in MSM, and ultimately to urge policy level changes to support disadvantaged populations.

In summary, our study suggests that we need to pay attention to feelings of loneliness and depressive symptoms and their closely associated factors such as social support and self-esteem among MSM in China. The MSM who were young, not married, and had low self-esteem were especially vulnerable to the impact of depressive symptoms on loneliness. Further research into the causes and consequences of mental health problems among Chinese MSM is necessary. Effective intervention programs addressing loneliness and negative emotions are needed to improve their psychological health. Providing a supportive community which embraces homosexuality with a tolerant attitude and eliminating discrimination against MSM may be the key to improve their living environment in China.

## Data availability statement

The raw data supporting the conclusions of this article will be made available by the authors, without undue reservation.

## Ethics statement

The studies involving human participants were reviewed and approved by the Ethics Committee of Taizhou Central Hospital. Written informed consent to participate in this study was provided by the participants’ legal guardian/next of kin.

## Author contributions

JW and XC conceived and designed the project. HL, TW, SW, and JK completed the questionnaire and collected the epidemiological data. JW, YL, and YY analyzed and interpreted the data. YL and YY prepared the manuscript. JW and CF reviewed the manuscript. All authors contributed to the article and approved the submitted version.
